# Supplementary material for: MetaQTL: a package of new computational methods for the meta-analysis of QTL mapping experiments
Source: BMC Bioinformatics. 2007 Feb 8;8:49. doi: 10.1186/1471-2105-8-49 (PMC1808479; doi:10.1186/1471-2105-8-49)
Supplement: Additional File 7 — MetaQTL Package : jar file and tutorial. This Zip archive contains both the MetaQTL JAR file and the files of the tutorial. [file 1471-2105-8-49-S7.zip › org.inra.metaqtl/doc/org/thalia/bio/IBioAdapter.html]

IBioAdapter


|  |  |  |
| --- | --- | --- |
| **Overview** \| **Package** \| **Class** \| **Use** \| **Tree** \| **Deprecated** \| **Index** \| **Help** | |  |
| **PREV CLASS**   **NEXT CLASS** | **FRAMES**    **NO FRAMES**     **All Classes** |  |
| SUMMARY: NESTED \| FIELD \| CONSTR \| METHOD | DETAIL: FIELD \| CONSTR \| METHOD |  |


---


## org.thalia.bio Interface IBioAdapter

**All Known Implementing Classes:**: BioEntityAdapter, BioEntityCollectionAdapter, GeneticMapBeanAdapter, LGroupBeanAdapter, LocusBeanAdapter, OntologyBeanAdapter, OntologyTermBeanAdapter

---

``` public interface IBioAdapter ```

---

| **Method Summary** | |
| --- | --- |
| `java.lang.Object` | `fromEntity(IBioEntity obj)`             Convert the given compliant object to a bio entity object. |
| `IBioEntity` | `toEntity(java.lang.Object obj)`             Convert the given bio entity object into another compliant object. |

**Method Detail**

### toEntity

```
IBioEntity toEntity(java.lang.Object obj)
```

:   Convert the given bio entity object into
    another compliant object.

    :   **Parameters:**: `obj` - **Returns:**

---


### fromEntity

```
java.lang.Object fromEntity(IBioEntity obj)
```

:   Convert the given compliant object to a
    bio entity object.

    :   **Parameters:**: `obj` - **Returns:**


---


|  |  |  |
| --- | --- | --- |
| **Overview** \| **Package** \| **Class** \| **Use** \| **Tree** \| **Deprecated** \| **Index** \| **Help** | |  |
| **PREV CLASS**   **NEXT CLASS** | **FRAMES**    **NO FRAMES**     **All Classes** |  |
| SUMMARY: NESTED \| FIELD \| CONSTR \| METHOD | DETAIL: FIELD \| CONSTR \| METHOD |  |


---
